# Determinant factors behind changes in health-seeking behaviour before and after implementation of universal health coverage in Indonesia

**DOI:** 10.1186/s12889-022-13142-8

**Published:** 2022-05-12

**Authors:** Dadan Mulyana Kosasih, Sony Adam, Mitsuo Uchida, Chiho Yamazaki, Hiroshi Koyama, Kei Hamazaki

**Affiliations:** 1 Department of Public Health, Graduate School of Medicine, Gunma University, Maebashi, Japan; 2 Health Office of Bandung City, Bandung, West Java Indonesia

**Keywords:** Universal health coverage, Health-seeking behaviour, Determinant factors, Indonesia

## Abstract

**Background:**

The health insurance system in Indonesia was transformed in 2014 to achieve universal health coverage (UHC). The effective implementation of essential primary health services through UHC has resulted in efficient healthcare utilisation, which is reflected in the health-seeking behaviour of the community. Our study aimed to examine the changes in health-seeking behaviour before and after the implementation of UHC in Indonesia and to identify what factors determine these changes.

**Methods:**

We conducted a retrospective cohort study using the recall method and data collected through questionnaire-based interviews in Bandung, Indonesia. We used a two-step sampling technique—randomised sampling and purposive sampling, and a total of 579 respondents with acute or chronic episodes were recruited. ${\chi }^{2}$ tests were used to identify the association between factors. Difference in difference model and a logistic regression model for binary outcomes were used to estimate the effect of the implementation of UHC on the health-seeking behaviour.

**Results:**

Utilisation of public health facilities increased significantly after implementation of UHC, from 34.9% to 65.4% among the respondents with acute episodes and 33.7% to 65.8% among those with chronic episodes. The odds of respondents going to health facilities when they developed an acute episode increased after the implementation of UHC (OR = 1.22, *p* = 0.05; AOR = 1.42, *p* < 0.001). For respondents experiencing chronic episodes, the implementation of UHC increased the odds ratio (OR = 1.74, *p* < 0.001; AOR = 1.64, *p* < 0.001) that they would use health facilities. Five years after the implementation of UHC, we still found respondents who did not have health insurance (26 and 19 respondents among those with acute episode and chronic episode, respectively).

**Conclusions:**

The effect of the implementation of UHC seemed greater for those experiencing chronic episodes than for those with an acute episode. Although the implementation of UHC has improved utilisation of public health facilities, the presence of people who are not covered by health insurance is a potential problem that could threaten future improvements in healthcare access and utilisation.

**Supplementary Information:**

The online version contains supplementary material available at 10.1186/s12889-022-13142-8.

## Background

Achieving universal health coverage (UHC) is one of the targets set by countries when they adopted the sustainable development goals in 2015 [1]. Since 2014, the Indonesian government has been running a national health insurance scheme, *Jaminan Kesehatan Nasional-Kartu Indonesia Sehat* (JKN-KIS), aimed at achieving UHC for all citizens by 2019 [2, 3]. JKN-KIS is organised under a mandatory social health insurance mechanism for all residents; thus, it potentially covers 100% of the population [4]. JKN-KIS merges Indonesia’s old insurance schemes, namely ASKES (*asuransi kesehatan*/ health insurance), JAMKESMAS (*jaminan kesehatan masyarakat*/ community health insurance), JAMSOSTEK (*jaminan sosial tenaga kerja*/ social labour security), and ASABRI (*asuransi sosial angkatan bersenjata Republik Indonesia*/ Indonesian armed forces social insurance) into a new health insurance scheme conducted by the Social Security Agency for Health (SSAH; or *Badan Penyelenggara Jaminan Sosial Kesehatan* [BPJS]). The SSAH has several unique features, including standards for staff performance and expertise, coverage goals and health objectives, and payment systems [5].

JKN-KIS participants generally consist of 1) contribution-assistance recipients (*peserta penerima bantuan iur*/ PBI), 2) wage-earning workers (*peserta pekerja penerima upah*/ PPU), 3) non-wage-earning workers (*peserta bukan penerima upah*/ PBPU), and 4) non-workers (*peserta bukan pekerja*) [6]*.* PBI is the poor and disadvantaged people, with the determination of participants in accordance with the provisions of the legislation. PPU covers every person who works for an employer by receiving a salary or wage, including civil servants, the army, the police, state officials, legislative members, non-civil servant government workers, private employees, and all other workers receiving a salary or wage. PBPU covers the self-employed, workers without a formal employment relationship, and all other workers not receiving a salary or wage. People who do not work but are able to pay a health insurance premium are considered non-workers. Non-workers include investors, employers, retired civil servants, war veterans, independence pioneers, widows, widowers, or orphans of war veterans or independence pioneers, and all other persons who are not working but are able to pay health insurance premiums [4, 6].

SSAH has collaborated with 16,831 first-level health facilities and 1,551 advanced level referral health facilities in 2014 [3]. These numbers increased to 23,145 first-level health facilities and 2,519 advanced level referral health facilities in 2019 [7]. By April 2018, JKN-KIS recorded as many as 196,662,064 participants, or 73.9% of the projected estimated population of Indonesia in 2018 [8–10], which is still far from the original target of as many as 235,100,000 participants JKN-KIS [11]. The number of residents who were not JKN-KIS participants was as high as 26.1% of the projected estimated population of Indonesia in 2018. In 2018, 44.3% of JKN-KIS participants were contribution-assistance recipients, 17.5% were wage-earning workers, 10.39% were non-wage-earning workers, and 1.9% were non-workers [8, 10].

The UHC efforts aim to meet several goals through prepayment schemes, often attempting to cross several hurdles in one leap [12]. The explicit aims are to guarantee access for everyone, to allow for the use of essential health services, and ensure that the use of these services does not expose the user to financial hardship [13]. The implicit aim that is rarely discussed, however, is that increasing access and utilisation rates for the formal health sector may reduce consumption of informal care, which is often inadequate, through self-medication or at-home treatment [12]. The effective implementation of essential primary healthcare services through UHC should result in efficient healthcare utilisation, which will reduce the disease burden and improve the overall well-being of the population [14]. Subsequently, because economic growth is directly related to improved health and well-being, UHC will improve the economic growth of the country [15].

Health care utilisation is directly related to the country's healthcare system and the health services that are provided [16, 17]. Meanwhile, the patterns of health-services utilisation are reflected in the health-seeking behaviours of the community. Thus, health services should be planned and provided based on information relating to health-seeking behaviours and utilisation of health services as well as their determining factors [18]. Andersen, in their most recent explication of the behavioural model of health services use, presented a conceptual framework that emphasises contextual and individual determinants of access to medical care [19]. The major components of the contextual and individual characteristics that determine access under the model are divided similarly, that is: predisposing factors, which are existing conditions that influence people to use or not use services; enabling factors, which are conditions that facilitate or hinder the use of services; and need factor, which is a condition recognised by laypeople or healthcare provider as requiring medical care [19, 20].

Underutilisation of health services is rarely due to the influence of local beliefs; rather it depends on the cost and availability of those services [21]. In developing countries, when people become ill, they usually try to cure themselves (especially for mild illnesses) using medicine advertised on television, radio, or newspapers; they will then seek medical treatment if the illness is not cured [22, 23]. In Indonesia, some patients will go to a traditional healer before they seek out health services [22]. In developing countries, healthcare professionals are relatively expensive, and prescription drugs are available as over-the-counter (OTC) drugs [23]. Such situations often lead to over-prescribing; delays in accessing rational, appropriate care within the formal health service; and can at times worsen the disease and increase mortality. This can ultimately result in higher treatment costs for the insurance scheme as the incidence of complex cases arriving at facilities may be higher than if patients accessed formal treatment at earlier stages of illness [12]. However, being covered by health insurance does not necessarily mean people will use their healthcare benefits. Based on an evaluation conducted by the Health Financing Centre of the Ministry of Health, it is known that about half of the covered people did not use the benefits for outpatient care, and 20% did not use the privileges for inpatient care [24].

Understanding healthcare-seeking behaviour and its determinants helps governments, stakeholders, policymakers, and healthcare providers to allocate and manage existing resources adequately, especially in developing countries [25–27]. Limited discussion exists on how the implementation of UHC affects people’s decisions to seek health care in Indonesia. In this study, we attempted to fill this information gap by examining the changes in health-seeking behaviour before and after implementation of UHC in Indonesia and exploring what factors determine these changes.

## Methods

### Study site

Bandung is one of the major cities in Indonesia, with an estimated population of about 2.5 million people in 2018 [28]. The city of Bandung has achieved UHC, with the proportion of insured people increasing from 66% in 2014 to 95% of the population as of 1 January 2018 [29]. Primary public health services are organised into 80 *puskesmas* (*pusat kesehatan masyarakat*/ public health centres) located in 30 sub-districts, such that in each sub-district, there are 1 to 5 *puskesmas* with distinct coverage areas.

### Study design

This was a retrospective cohort study using a recall method. We used this method because the UHC has been implemented since 2014. We collected the data between July and August 2019 to assess changes in health-seeking behaviours before and after the implementation of UHC through an interview questionnaire. The interview was administered by *puskesmas* health workers in 30 sub-districts of Bandung city.

We realised that the limitation of the retrospective cohort study is that it relies on the accuracy of individual recall. Therefore, to minimise bias that might occur due to errors in recruiting respondents or the possibility of respondents forgetting, how the data was obtained is very important [30]. We trained the interviewers so that they were able to obtain the past time information from the respondents. We ensured that the interviewers had the same understanding about the research and how to conduct interviews to gather respondents’ information and fill in the obtained information in the questionnaire correctly.

Sample size was estimated using EZR’s ‘sample size calculation for comparison with a specified proportion’ [31]. The sample size was estimated in advance to have 80% power of detecting a change in health-seeking behaviour among residents before and after UHC implementation, assuming a 2-sided Type 1 error probability of 0.05. Additionally, given the results of the data on healthcare utilisation trends from the Health Policy Plus and National Team for the Acceleration of Poverty Reduction Indonesia 2011–2016, we assume a proportion control of 11% as well as a proportion test of 15% [32]. It was estimated that a sample size of 572 would be sufficient to detect the differences in health-seeking behaviour between the residents before and after the implementation of UHC. The number of samples was then proportionally divided into 30 sub-districts in Bandung according to the population in each sub-district, resulting in 6 to 31 samples in each sub-district. The number of sub-district samples was then also divided by the number of *puskesmas* in the sub-district, according to the population in the work area of each *puskesmas*.

**Fig. 1:**
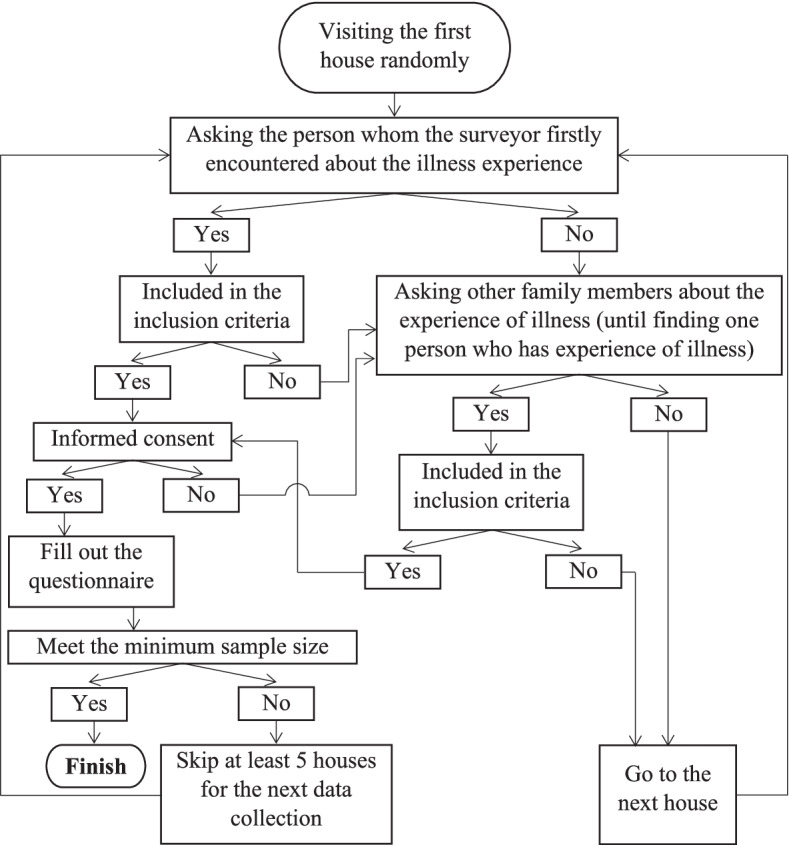
Data collection pathway

Respondents were recruited using a two-step sampling technique—randomised sampling and purposive sampling with the following criteria: resident of Bandung, aged 23 years or older, and experienced recent illness (acute illness in the previous two weeks and/or chronic disease). Each respondent came from a different household, and each household had an interval of at least five houses from other participating households. The questionnaires were filled in at the respondent’s homes, which were located in the coverage area of the surveyor’s *puskesmas*, following the data collection path shown in Fig. 1.

At the time of data collection, the surveyor visited the respondents’ houses at random. The surveyor asked the first person they met in the house if they had ever been sick. If the answer was ‘YES’, the process continued to check the inclusion criteria, informed consent, and filled out the questionnaire. After completion, the next data collection was carried out at the next house, which was at least 5 houses apart from the house where the previous data had been gathered. If the answer to the question of whether they had ever been sick was ‘NO’, the surveyor would ask other family members who lived in the house if they had ever been sick. If the answer was ‘YES’, the process continued, and the surveyor checked the inclusion criteria, obtained informed consent, and filled out the questionnaire. If none of the other family members living in the house had experienced illness, the surveyor moved on to the next house. This data collection process continued until the minimum number of samples were collected.

### Survey Instrument

The questionnaire contained demographic and socio-economic information, including sex, age, education, occupation, marital status, personal and household income, and information regarding health insurance ownership. It also included a set of questions regarding health-seeking behaviour. We asked questions about whether the respondents had developed an acute episode and/or chronic episode after the implementation of UHC (2014–2019); then, we asked about their experience with similar acute episodes and/or chronic episodes before the implementation of UHC (2009–2013). The questions about acute and chronic episodes were then followed by different serial questions. These questions asked about their experience in seeking care when they were sick before and after UHC implementation and whether they sought care outside their home. If they sought care for acute episodes, respondents were asked where they first received it; if they were sought/were seeking care for chronic episodes, they were asked to name the facility they went to most often. The questionnaire was developed in reference to existing tools that have been used in related studies [33, 34].

### Variables

#### Dependent variables

We utilised health-seeking behaviours as the outcome variable. We defined health-seeking behaviours as any action undertaken by individuals who perceive themselves to have a health problem or to be ill to find an appropriate remedy [21]. We classified health-seeking behaviour into four categories: 1) no medication, 2) informal care, 3) public health facility, and 4) private health facility. ‘No medication’ indicated that the people reported experiencing perceived illness but did not use any health services or medications. ‘Informal care’ was defined as the use of any facility that was not in a public or private health facility. Specifically, the use of traditional or over-the-counter drugs and the use of traditional healers were included in this category. ‘Public health facility’ indicated that the people were using a health facility that is owned and managed by the government. ‘Private health facility’ indicated that the people used health services in a facility not owned and managed by the government. We set the dependent variable as a binary variable according to the action that was taken by respondents when they developed an illness. In this study, the description of the variable is taken as a value 1 if a respondent chose health facilities including public and private health facilities. On the contrary, the description of the variable is taken as the value 0 if a respondent chose a non-health facility, such as no medication and informal care.

#### Independent variables

The implementation of UHC was involved as an essential independent variable. The implementation of UHC = 1 means that the respondent is having health insurance, and the year is after UHC implementation period (2014–2019); otherwise, the implementation of UHC = 0.

#### Control variables

In line with the existing literature, we grouped the control variables that might influence the health seeking behaviour based on Andersen’s behavioural model of health services use (see Supplementary Table 1 for the variable definitions). We included age, sex, marital status, education, and employment status as predisposing factors. Adjusted household income, change in insurance ownership (before and after UHC implementation), and type of health insurance membership (JKN-KIS membership category) were included as enabling factors. We used the perception of the seriousness of acute illness (for respondents with acute episode), number of chronic diseases (for respondents with chronic episode), perception of general health conditions, and change in health status before and after UHC implementation as need factors.

### Data Analysis

Since the implementation of the UHC in Indonesia began in 2014, we set 2009 to 2013 as the period before the implementation of UHC and 2014 to 2019 as the period after the implementation of UHC. By using Pearson’s chi-squared tests, we analysed respondents with acute and chronic episodes separately. The health-seeking behaviour of the respondents in the period of 2009 to 2013 was then compared to the period of 2014 to 2019 in each illness episode.

The difference-in-differences (DID) method is key to assessing interventions to advise health policymakers and future policies [35]. The DID method can unravel the impact of the intervention from the permanent differences between the intervention group and the control group and the temporal trends of results that are not related effectively to the implementation of UHC. The effect of the implementation of UHC is estimated by comparing the differences between two changes in outcomes, firstly, changes between the pre- and post-implementation UHC periods within the insured people group (implemented UHC) and secondly, the pre- and post-implementation UHC periods in the uninsured group (unimplemented UHC). We employed the DID model and used a logistic regression model for binary outcomes to estimate the effect of the implementation of UHC on the health-seeking behaviour in each of the samples. We adjusted results for several potential confounders including: age, sex, marital status, education, employment status, adjusted household income, change in insurance ownership (before and after UHC implementation), type of health insurance membership (JKN-KIS membership category), the perception of the seriousness of acute illness (for respondents with acute episode), number of chronic diseases (for respondents with chronic episode), perception of general health conditions, and change in health status before and after UHC implementation. The regression model can be presented with Eq. (1) [36]:

1 $${y}_{ist}={A}_{s}+{B}_{t}+c{X}_{ist}+\beta {I}_{ist}+{\varepsilon }_{ist}$$

In Eq. (1), ${y}_{ist}$ is a measure of an individual $i$ of the group $s$ in the year $t$, ${A}_{s}$ is the treatment/control group fixed-effect, ${B}_{t}$ is before/after for a year fixed-effect, $c{X}_{ist}$ is a set of individual-level control variables and ${\varepsilon }_{ist}$ is the error term. The variable of interest, ${I}_{ist}$, is a dummy variable that equals one with regards to the years after the implementation of UHC and equals zero otherwise. The key parameter of interest is *β*, the DID estimate, which measures the pre-post change in health-seeking behaviour, thereby indicating the effect of the implementation of UHC on health-seeking behaviour. A value of greater than 1 and significant *β* suggests that the implementation of UHC has a positive effect on guiding respondents to health facilities for health services and vice versa. The DID estimation approach allows us to control for omitted variables. Statistical analyses were carried out using RStudio version 1.4.1717.

## Results

**Fig. 2:**
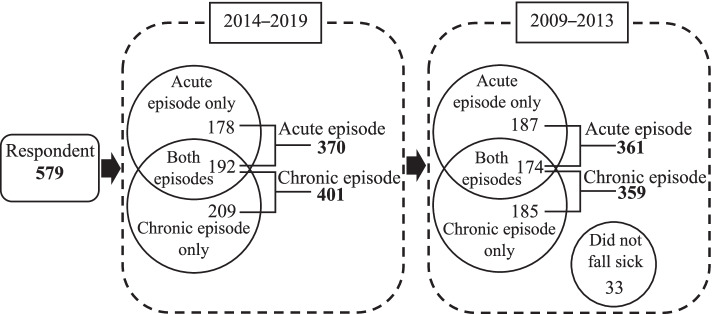
Number of samples

We visited 658 houses, and a total of 582 participants were enrolled in the survey for an 88.44% response rate. Of these, three were excluded, leaving a total study population of 579 respondents. Among the 579 respondents, we obtained 370 respondents who had an acute episode, and 401 respondents had a chronic episode during the of 2014–2019 period. From these numbers, 361 respondents had an acute episode, and 359 respondents had a chronic episode during the of 2009–2013 period (see Fig. 2).

### Respondents with acute episodes

Table 1 presents the distribution of health-seeking behaviour that the respondents chose when they had an acute illness; the table is organised by socio-demographic characteristics, before the implementation of UHC (2009–2013) and after the implementation of UHC (2014–2019).

**Table 1:** Acute episode health-seeking behaviour: Individual characteristics of respondents

Variable	2009–2013											2014–2019											Comparison of the 2009-2013 and the 2014-2019 factors
	Total		No Medication		Informal care		Public Health Facility		Private Health Facility		*p*-value*	Total		No Medication		Informal care		Public Health Facility		Private Health Facility		*p*-value*	
	*n*	%	*n*	%	*n*	%	*n*	%	*n*	%		*n*	%	*n*	%	*n*	%	*n*	%	*n*	%		*p*-value*
Total	361	100	103	28.6	86	23.8	126	34.9	46	12.7		370	100	53	14.3	23	6.2	242	65.4	52	14.1		<0.001
**Predisposing factors**																							
Age																							
23-39	109	100	37	33.9	38	34.9	22	20.2	12	11	<0.001	110	100	21	19.1	12	10.9	58	52.7	19	17.3	0.03	<0.001
40-59	166	100	49	29.5	30	18.1	68	41	19	11.4		171	100	19	11.1	7	4.1	123	71.9	22	12.9		<0.001
> 60	86	100	17	19.8	18	20.9	36	41.9	15	17.4		89	100	13	14.6	4	4.5	61	68.5	11	12.4		<0.001
Sex																							
Male	97	100	28	28.9	27	27.8	28	28.9	14	14.4	0.46	99	100	17	17.2	6	6.1	60	60.5	16	16.2	0.64	<0.001
Female	264	100	75	28.4	59	22.3	98	37.1	32	12.1		271	100	36	13.3	17	6.3	182	67.1	36	13.3		<0.001
Education																							
Primary school or below	87	100	16	18.4	16	18.4	45	51.7	10	11.5	0.01	88	100	12	13.6	3	3.4	68	77.3	5	5.7	0.04	<0.001
Junior or senior high school	191	100	63	33	46	24.1	58	30.4	24	12.5		197	100	24	12.2	14	7.1	127	64.5	32	16.2		<0.001
College or above	83	100	24	28.9	24	28.9	23	27.7	12	14.5		85	100	17	20	6	7.1	47	55.3	15	17.6		<0.001
Occupation																							
Unemployed	178	100	51	28.7	36	20.2	69	38.8	22	12.4	0.45	184	100	21	11.4	10	5.4	129	70.1	24	13	0.25	<0.001
Civil Servant	48	100	17	35.4	13	27.1	13	27.1	5	10.4		49	100	13	26.5	2	4.1	27	55.1	7	14.3		0.002
Private Employed	41	100	13	31.7	10	24.4	10	24.4	8	19.5		43	100	7	16.3	5	11.6	22	51.2	9	20.9		0.04
Self-employed	72	100	17	23.6	22	30.6	27	37.5	6	8.3		72	100	10	13.9	4	5.6	50	69.4	8	11.1		<0.001
Retired	22	100	5	22.7	5	22.7	7	31.8	5	22.7		22	100	2	9.1	2	9.1	14	63.6	4	18.2		0.18
Marital status																							
Married	279	100	83	29.7	68	24.4	90	32.3	38	13.6	0.2	286	100	35	12.2	20	7	188	65.7	43	15	0.02	<0.001
Never married	23	100	8	34.8	7	30.4	7	30.4	1	4.3		23	100	10	43.5	1	4.3	10	43.5	2	8.7		0.13
Divorced/ Widowed/ Separated	59	100	12	20.9	11	18.6	29	49.2	7	11.9		61	100	8	13.1	2	3.3	44	72.1	7	11.5		0.01
Variable	2009–2013											2014–2019											Comparison of the 2009-2013 and the 2014-2019 factors
	Total		No Medication		Informal care		Public Health Facility		Private Health Facility		p-value*	Total		No Medication		Informal care		Public Health Facility		Private Health Facility		p-value*	
	n	%	n	%	n	%	n	%	n	%		n	%	n	%	n	%	n	%	n	%		p-value*
**Enabling factors**																							
Adjusted household income																							
0 – 2,999,999 IDR (0 – 211.78 USD)	293	100	79	27	69	23.5	108	36.9	37	12.6	0.36	302	100	39	12.9	20	6.7	204	67.5	39	12.9	0.21	<0.001
3,000,000 – 5,999,999 IDR (211.79 – 423.56 USD)	62	100	23	37.1	14	22.6	16	25.8	9	14.5		62	100	14	22.6	3	4.8	33	53.2	12	19.4		0.001
> 6,000,000 IDR (> 423. 57 USD)	6	100	1	16.7	3	50	2	33.3	0	0		6	100	0	0	0	0	5	83.3	1	16.7		0.1
Health insurance ownership																							
Did not have health insurance	203	100	63	31	44	21.7	72	35.5	24	11.8	0.97	26	100	6	23.1	8	30.8	6	23.1	6	23.1	<0.001	0.2
Social health insurance	129	100	33	25.6	34	26.4	44	34.1	18	14		316	100	43	13.6	13	4.1	222	70.3	38	12		<0.001
Private health insurance	25	100	6	24	7	28	9	36	3	12		0	0	0	0	0	0	0	0	0	0		1
Private and social health insurance	4	100	1	25	1	25	1	25	1	25		28	100	4	14.3	2	7.1	14	50	8	28.6		0.43
Type of JKN-KIS (national health insurance) membership																							
Not a JKN-KIS member	25	100	13	52	8	32	0	0	4	16	0.08	26	100	6	20.1	8	30.8	6	23.1	6	23.1	<0.001	0.03
Wage-earning workers	76	100	21	27.6	21	27.6	23	30.3	11	14.5		77	100	15	19.5	4	5.2	43	55.8	15	19.5		<0.001
Contribution-assistance recipients	183	100	47	25.7	39	21.3	76	41.5	21	11.5		188	100	21	11.2	7	3.7	143	76.1	17	9		<0.001
Non-wage-earning workers	59	100	17	28.8	14	23.7	21	35.6	7	11.9		61	100	10	16.4	2	3.3	39	63.9	10	16.4		<0.001
Non-workers	18	100	5	27.8	4	22.2	6	33.3	3	16.7		18	100	1	5.6	2	11.1	11	61.1	4	22.2		0.19
Variable	2009-2013											2014-2019											Comparison of the 2009-2013 and the 2014-2019 factors
	Total		No Medication		Informal care		Public Health Facility		Private Health Facility		p-value*	Total		No Medication		Informal care		Public Health Facility		Private Health Facility		p-value*	
	n	%	n	%	n	%	n	%	n	%		n	%	n	%	n	%	n	%	n	%		p-value*
Coverage changes																							
Always covered	156	100	40	25.6	41	26.3	54	34.6	21	13.5	0.02	159	100	25	15.7	7	4.4	99	62.3	28	17.6	<0.001	<0.001
Gained coverage	180	100	50	27.8	37	20.6	72	40	21	11.7		185	100	22	11.9	8	4.3	137	74.1	18	9.7		<0.001
Lost coverage	2	100	0	0	1	50	0	0	1	50		2	100	0	0	1	50	0	0	1	50		1
Never covered	23	100	13	56.5	7	30.5	0	0	3	13		24	100	6	25	7	29.2	6	25	5	20.8		0.02
**Need factors**																							
Seriousness																							
Not serious	185	100	64	34.6	49	26.5	49	26.5	23	12.4	0.02	188	100	38	20.2	16	8.5	113	60.1	21	11.2	0.01	<0.001
Somewhat serious	162	100	34	21	35	21.6	70	43.2	23	14.2		168	100	14	8.3	7	4.2	119	70.8	28	16.7		<0.001
Serious	14	100	5	35.7	2	14.3	7	50	0	0		14	100	1	7.1	0	0	10	71.5	3	21.4		0.04
Current health status																							
Fair or poor	131	100	36	27.5	26	19.8	53	40.5	16	12.2	0.18	137	100	17	12.4	6	4.4	94	68.6	20	14.6	0.03	<0.001
Good	213	100	59	27.7	54	25.4	71	33.3	29	13.6		216	100	29	13.4	17	7.9	141	65.3	29	13.4		<0.001
Very good or excellent	17	100	8	47.1	6	35.2	2	11.8	1	5.9		17	100	7	41.2	0	0	7	41.2	3	17.6		0.02
Health status change																							
Worse	27	100	8	29.6	3	11.1	12	44.4	4	14.8	0.25	27	100	1	3.7	1	3.7	20	74.1	5	18.5	0.01	0.03
Same	207	100	63	30.4	57	27.5	63	30.4	24	11.6		213	100	36	16.9	22	10.3	124	58.2	31	14.6		<0.001
Better	127	100	32	25.2	26	20.5	51	40.2	18	14.2		130	100	16	12.3	0	0	98	75.4	16	12.3		<0.001

*IDR* Indonesian Rupiah, *USD* US dollar

*: Pearson’s Chi-squared test

Of respondents with acute episodes before the implementation of UHC, 28.6% chose no medication, 23.8% chose informal care, 34.9% chose a public health facility, and 12.7% chose a private health facility. Age, education, coverage change, and seriousness of the illness were significantly associated with health-seeking behaviour before the implementation of UHC. After the implementation of UHC, the proportion of respondents who chose no medication and informal care decreased to 14.3% and 6.2%, respectively. The proportion of respondents who chose public health facilities and private health facilities increased to 65.4% and 14.1%, respectively. Age, education, marital status, health insurance ownership, type of JKN-KIS membership, coverage change, seriousness of the illness, current health status, and health status change were significantly associated with health-seeking behaviour after the implementation of UHC.

### Determinant factors related to change in health-seeking behaviour

#### Predisposing factors

All predisposing factors examined in this study were significantly associated with changes in health-seeking behaviour by the respondents that experienced acute episodes, except for those who were retired or were never married. It is worth noting, if we look further, among those who were never married, the proportion of respondents who chose no medication increased from 34.8% to 43.5%.

#### Enabling Factors

Adjusted household income was significantly associated with changes in the health-seeking behaviour of the respondents that experienced acute episodes, except those who had high adjusted household incomes. The type of JKN-KIS membership was also significantly associated with changes in health-seeking behaviour, except for those who were included as non-workers. In the health insurance ownership variable, social insurance was the only factor that had a significant association with changes in health-seeking behaviour. We found that 26 respondents did not have health insurance after the implementation of UHC. Of those, two respondents had lost their coverage, and 24 respondents were never covered by health insurance in either period.

#### Need factors

All the need factors examined in this study were significantly associated with changes in health-seeking behaviour of the respondents that experienced acute episodes.

### Respondents with chronic episodes

Table 2 presents the distribution of health-seeking behaviour that the respondents chose when they had chronic episodes, organised by socio-demographic characteristics, before the implementation of UHC (2009–2013) and after the implementation of UHC (2014–2019).

**Table 2:** Chronic episode health-seeking behaviour: Individual characteristics of respondents

Variable	2009-2013											2014-2019											Comparison of the2009-2013 and the 2014-2019 factors
	Total		No Medication		Informal care		Public Health Facility		Private Health Facility		*p*-value*	Total		No Medication		Informal care		Public Health Facility		Private Health Facility		*p*-value*	
	*n*	%	*n*	%	*n*	%	*n*	%	*n*	%		*n*	%	*n*	%	*n*	%	*n*	%	*n*	%		*p*-value*
Total	359	100	107	29.8	69	19.2	121	33.7	62	17.3		401	100	20	5	16	4	264	65.8	101	25.2		<0.001
**Predisposing factors**																							
Age (years)																							
23-39	69	100	23	33.3	25	36.2	13	18.8	8	11.7	<0.001	81	100	4	4.9	8	9.9	52	64.2	17	21	0.13	<0.001
40-59	161	100	51	31.7	34	21.1	51	31.7	25	15.5		182	100	10	5.5	4	2.2	121	66.5	47	25.8		<0.001
> 60	129	100	33	25.6	10	7.8	57	44.2	29	22.5		138	100	6	4.3	4	2.9	91	65.9	37	25.8		<0.001
Sex																							
Male	90	100	23	25.6	17	18.9	30	33.3	20	22.2	0.48	101	100	2	2	4	4	66	65.3	29	28.7	0.37	<0.001
Female	269	100	84	31.2	52	19.3	91	33.8	42	15.6		300	100	18	6	12	4	198	66	72	24		<0.001
Education																							
Primary school or below	112	100	29	25.9	17	15.2	49	43.7	17	15.2	0.14	120	100	10	8.3	1	0.8	90	75	19	15.8	<0.001	<0.001
Junior or senior high school	174	100	51	29.3	37	21.3	55	31.6	31	17.8		200	100	8	4	12	6	134	67	46	23		<0.001
College or above	73	100	27	37	15	20.5	17	23.3	14	19.2		81	100	2	2.5	3	3.7	40	49.4	36	44.4		<0.001
Occupation																							
Unemployed	194	100	55	28.4	38	19.6	73	37.6	28	14.4	0.16	215	100	15	7	10	4.7	139	64.7	51	23.7	0.25	<0.001
Civil Servant	40	100	15	37.5	6	15	12	30	7	17.5		44	100	1	2.3	2	4.5	27	61.4	14	31.8		<0.001
Private Employed	35	100	13	37.1	7	20	7	20	8	22.9		37	100	0	0	1	2.7	26	70.3	10	27		<0.001
Self-employed	61	100	17	27.9	16	26.2	14	23	14	23		72	100	4	5.6	1	1.4	54	75	13	18.1		<0.001
Retired	29	100	7	24.1	2	6.9	15	51.7	5	17.2		33	100	0	0	2	6.1	18	54.5	13	39.4		0.008
Marital status																							
Married	257	100	83	32.3	50	19.5	79	30.7	45	17.5	0.27	285	100	15	5.3	12	4.2	185	64.9	73	25.6	0.13	<0.001
Never married	19	100	5	26.3	6	31.6	6	31.6	2	10.5		22	100	0	0	3	13.6	16	72.7	3	13.6		0.01
Divorced/ Widowed/ Separated	83	100	19	22.9	13	15.7	36	43.4	15	18		94	100	5	5.3	1	1.1	63	67	25	26.6		<0.001
Variable	2009-2013											2014-2019											Comparison of the2009-2013 and the 2014-2019 factors
	Total		No Medication		Informal care		Public Health Facility		Private Health Facility		p-value*	Total		No Medication		Informal care		Public Health Facility		Private Health Facility		p-value*	
	n	%	n	%	n	%	n	%	n	%		n	%	n	%	n	%	n	%	n	%		p-value*
**Enabling factors**																							
Adjusted household income																							
0 – 2,999,999 IDR (0 – 211.78 USD)	293	100	89	30.4	58	19.8	103	35.2	43	14.6	0.02	324	100	18	5.6	10	3.1	221	68.2	75	23.1	0.04	<0.001
3,000,000 – 5,999,999 IDR (211.79 – 423.56 USD)	52	100	15	28.8	11	21.2	14	26.9	12	23.1		60	100	2	3.3	6	10	32	53.3	20	33.3		<0.001
> 6,000,000 IDR (> 423. 57 USD)	14	100	3	21.4	0	0	4	28.6	7	50		17	100	0	0	0	0	11	64.7	6	35.3		0.04
Health insurance ownership																							
Did not have health insurance	182	100	60	33	43	23.6	54	29.7	25	13.7	0.01	19	100	1	5.3	4	21.1	4	21.1	10	52.6	<0.001	<0.001
Social health insurance	155	100	41	26.5	19	12.3	66	42.6	29	18.7		345	100	15	4.3	10	2.9	244	70.7	76	22		<0.001
Private health insurance	18	100	6	33.3	6	33.3	0	0	6	33.3		3	100	0	0	1	33.3	1	33.3	1	33.3		0.21
Private and social health insurance	4	100	0	0	1	25	1	25	2	50		34	100	4	11.8	1	2.9	15	44.1	14	41.2		0.35
Type of JKN-KIS (national health insurance) membership																							
Not a JKN-KIS member	19	100	4	21.1	5	26.9	2	10.5	8	42.1	<0.001	22	100	1	4.5	5	22.7	5	22.7	11	50	<0.001	0.39
Wage-earning workers	75	100	26	34.7	12	16	18	24	19	25.3		81	100	5	6.2	5	6.2	42	51.9	29	35.8		<0.001
Contribution-assistance recipients	181	100	57	31.5	29	16	71	39.2	24	13.3		199	100	12	6	3	1.5	155	77.9	29	14.6		<0.001
Non-wage-earning workers	56	100	13	23.2	20	35.7	17	30.4	6	10.7		67	100	2	3	1	1.5	45	67.2	19	28.4		<0.001
Non-workers	28	100	7	25	3	10.7	13	46.4	5	17.9		32	100	0	0	2	6.2	17	53.1	13	40.6		0.006
Variable	2009-2013											2014-2019											Comparison of the2009-2013 and the 2014-2019 factors
	Total		No Medication		Informal care		Public Health Facility		Private Health Facility		p-value*	Total		No Medication		Informal care		Public Health Facility		Private Health Facility		p-value*	
	n	%	n	%	n	%	n	%	n	%		n	%	n	%	n	%	n	%	n	%		p-value*
Coverage changes																							
Always covered	176	100	47	26.7	26	14.8	67	38	36	20.5	0.01	195	100	6	3.1	8	4.1	130	66.7	51	26.2	<0.001	<0.001
Gained coverage	167	100	56	33.5	40	24	52	31.1	19	11.4		187	100	13	7	4	2.1	130	69.5	40	21.4		<0.001
Lost coverage	1	100	0	0	0	0	0	0	1	100		1	100	0	0	0	0	0	0	1	100		1
Never covered	15	100	4	26.7	3	20	2	13.3	6	40		18	100	1	5.6	4	22.2	4	22.2	9	50		0.44
**Need factors**																							
Number of chronic diseases																							
1	201	100	70	34.8	52	25.9	50	24.9	29	14.4	<0.001	233	100	12	5.2	12	5.2	161	69.1	48	20.6	0.09	<0.001
2	97	100	24	24.7	9	9.3	46	47.4	18	18.6		105	100	6	5.7	1	1	68	64.8	30	28.6		<0.001
3 or more	61	100	13	21.3	8	13.1	25	41	15	24.6		63	100	2	3.2	3	4.8	35	55.6	23	36.5		0.003
Current health status																							
Fair or poor	150	100	41	27.3	21	14	59	39.3	29	19.3	0.03	163	100	8	4.9	2	1.2	109	66.9	44	27	0.27	<0.001
Good	201	100	61	30.3	45	22.4	62	30.8	33	16.4		229	100	11	4.8	13	5.7	149	65.1	56	24.5		<0.001
Very good or excellent	8	100	5	62.5	3	37.5	0	0	0	0		9	100	1	11.1	1	11.1	6	66.7	1	11.1		0.009
Health status change																							
Worse	36	100	8	22.2	5	13.9	15	41.7	8	22.2	0.47	38	100	2	5.3	0	0	26	68.4	10	26.3	0.47	0.006
Same	205	100	64	31.3	40	19.5	62	30.2	39	19		235	100	12	5.1	12	5.1	146	62.1	65	27.7		<0.001
Better	118	100	35	29.7	24	20.3	44	37.3	15	12.7		128	100	6	3.1	4	3.1	92	71.9	26	20.3		<0.001

*IDR* Indonesian Rupiah, *USD* US dollar

*: Pearson’s Chi-squared test

Of the respondents with chronic episodes before the implementation of UHC, 29.8% chose no medication, 19.2% chose informal care, 33.7% chose a public health facility, and 17.3% chose a private health facility. Age, adjusted household income, health insurance ownership, type of JKN-KIS membership, coverage change, the number of chronic diseases, and current health status were significantly associated with health-seeking behaviour before the implementation of UHC. After the implementation of UHC, the proportion of respondents who chose no medication and informal care decreased to 5.0% and 4.0%, respectively. The proportion of respondents who chose public health facilities and private health facilities increased to 65.8% and 25.2%, respectively. Education, adjusted household income, health insurance ownership, type of JKN-KIS membership, and coverage change were significantly associated with health-seeking behaviour after the implementation of UHC.

### Determinant factors related to change in health-seeking behaviour

#### Predisposing factors

Among the respondents with chronic episodes, all predisposing factors examined in this study were significantly associated with changes in health-seeking behaviour.

#### Enabling factors

Adjusted household income was significantly associated with changes in the health-seeking behaviour of respondents with chronic episodes. The type of JKN-KIS membership was also significantly associated with changes in health-seeking behaviour, except for those who were not JKN-KIS members. In the health insurance ownership variable, health insurance and social insurance were not significantly associated with changes in health-seeking behaviour. We found that 19 respondents did not have health insurance after the implementation of UHC. Of those, one respondent lost their coverage, and 18 respondents were never covered by health insurance in either period.

#### Need factors

All the need factors examined in this study were significantly associated with changes in the health-seeking behaviour of the respondents with chronic episode.

### Difference in difference estimates

The majority of respondents were female, young adults to middle age, graduated from junior or senior high school, married, had low adjusted household incomes, had a good current health condition, and they thought that they had the same health condition before and after implementation of UHC (see Supplementary Table 2). It was true for those who had health insurance and those who did not have insurance after the implementation of UHC. Regarding the occupation, the majority of those who did not have health insurance after the implementation of UHC were unemployed and those who worked as private employed. In contrast, those who had health insurance after the implementation of UHC were mainly civil servants.

Table 3 describes the results of DID analysis, which determine the effects of the implementation of UHC on respondents’ health seeking behaviour. There were no control variables included in model 1. DID with predisposing, enabling, and need covariates in model 2 was employed to assess the effect of the implementation of UHC on respondents’ health-seeking behaviour. As shown in Table 3, for respondents with an acute episode, the odds ratio of the implementation of UHC was above 1 but not significant statistically in model 1. However, after controlling for the variables of predisposing, enabling, and need in model 2, the odds ratio of the implementation of UHC was above 1 and significant statistically. That is to say, the odds of respondents going to health facilities when they developed an acute episode after the implementation of UHC was 42%, which was higher than before the implementation of UHC (OR = 1.22, *p* = 0.05, in model 1; AOR = 1.42, *p* < 0.001, in model 2), which indicated that implementation of UHC had a significantly positive effect on the likelihood of guiding respondents with an acute episode to health facilities for contact. In other words, respondents with acute episodes were significantly more likely to go to health facilities after the implementation of UHC. With regards to respondents with chronic episodes, all the odds ratios of UHC were also above 1 but significant statistically upon both models (AOR = 1.74, *p* < 0.001, in model 1; AOR = 1.64, *p* < 0.001, in model 2), which suggested that the effect of the implementation of UHC was significantly positive on the likelihood of guiding respondents with chronic episodes to health facilities for contact.

**Table 3:** Effect of the implementation of UHC on health seeking behaviour

	Acute episode								Chronic episode							
	Model1				Model2				Model1				Model2			
	Odds Ratios	95%CI		*p*-value	Adjusted Odds Ratios	95%CI		*p*-value	Odds Ratios	95%CI		*p*-value	Adjusted Odds Ratios	95%CI		*p*-value
Implementation of UHC	1.22	1.00	1.50	0.05	1.42	1.16	1.74	<0.001	1.74	1.45	2.08	<0.001	1.64	1.36	1.98	<0.001
**Predisposing factors**																
Age																
40-59					1.13	1.04	1.23	<0.01					1.06	0.96	1.15	0.24
> 60					1.10	0.98	1.24	0.15					1.15	1.03	1.28	<0.05
Sex																
Female					0.97	0.89	1.06	0.48					1.03	0.95	1.11	0.49
Education																
Junior or senior high school					0.97	0.89	1.06	0.44					1.00	0.93	1.08	0.93
College or above					1.01	0.89	0.89	0.89					0.98	0.87	1.09	0.67
Occupation																
Civil Servant					0.92	0.80	1.05	0.18					1.06	0.93	1.20	0.39
Private Employed					1.04	0.92	1.17	0.70					1.07	0.95	1.20	0.28
Self-employed					1.01	0.92	1.11	0.79					1.01	0.93	1.10	0.83
Retired					1.13	0.82	1.56	0.47					1.11	0.89	1.39	0.36
Marital status																
Never married					0.90	0.78	1.04	0.15					1.03	0.89	1.18	0.71
Divorced/ Widowed/ Separated					1.03	0.93	1.14	0.58					1.02	0.94	1.10	0.63
**Enabling factors**																
Adjusted household income																
3,000,000 – 5,999,999 IDR (211.79 – 423.56 USD)					0.97	0.87	1.08	0.55					1.02	0.93	1.12	0.69
> 6,000,000 IDR (> 423. 57 USD)					1.25	0.95	1.63	0.11					1.25	1.07	1.47	<0.01
Health Insurance Ownership																
Having health insurance					1.02	0.93	1.11	0.65					1.07	0.98	1.16	0.12
Type of JKN-KIS (national health insurance) membership																
Wage-earning workers					1.37	1.16	1.63	<0.001					1.22	0.86	1.72	0.26
Contribution-assistance recipients					1.36	1.17	1.58	<0.001					1.29	0.92	1.82	0.14
Non-wage-earning workers					1.37	1.16	1.61	<0.001					1.23	0.87	1.75	0.24
Non-workers					1.19	0.81	1.75	0.50					1.09	0.73	1.62	0.69
Coverage changes																
Gained coverage					0.90	0.58	1.41	0.65					1.86	0.97	3.55	0.06
Lost coverage					0.94	0.86	1.03	0.18					1.02	0.94	1.10	0.65
Never covered					0.73	0.63	0.85	<0.001					1.21	0.82	1.77	0.34
**Need factors**																
Seriousness of acute illness																
Somewhat serious					1.12	1.04	1.20	<0.01								
Serious					1.10	0.93	1.31	0.30								
Number of chronic diseases																
2													1.11	1.04	1.19	<0.01
3 or more													1.08	0.99	1.17	0.08
Current health status																
Good					1.05	0.97	1.13	0.23					0.95	0.89	1.01	0.12
Very good or excellent					0.84	0.71	0.99	<0.05					0.76	0.62	0.94	<0.01
Health status change																
Same					0.90	0.79	1.03	0.10					0.98	0.88	1.08	0.68
Better					0.99	0.87	1.14	0.82					0.97	0.85	1.04	0.55

Table 3 also characterises the association between influence factors and health-seeking behaviour. As observed from Table 3 the odds of respondents with acute and chronic episodes, with very good or excellent current health status, choosing health facilities were 16% and 24.0% lower than residents with fair or poor current health status (AOR = 0.84, *p* < 0.05; AOR = 0.76, *p* < 0.015, respectively), which indicated that very good or excellent current health had a significantly negative effect on the probability of guiding respondents with acute or chronic episodes to go to health facilities. Moreover, the likelihood of respondents with acute episodes of somewhat serious illness choosing health facilities were 1.12 times that of respondents with acute episode with no serious illness (AOR = 1.12, *p* < 0.01), which suggested that respondents with acute episodes of somewhat serious illness were significantly more likely to go to health facilities compared to those with no serious illness. For respondents with chronic episodes, those diagnosed with 2 chronic diseases were significantly more likely to go to health facilities—1.11 times higher than respondents with only 1 chronic disease diagnosis (AOR = 1.11, *p* < 0.01). For respondents with acute episodes, being in middle adulthood, including wage-earning workers, contribution-assistance recipients, and non-wage-earning workers with JKN-KIS membership, had a positive effect. This indicated that respondents in middle adulthood were significantly more likely to go to health facilities compared to those in early adulthood. JKN-KIS members including wage-earning workers, contribution-assistance recipients, and non-wage-earning workers were significantly more likely to go to health facilities than non-JKN-KIS members. Never being covered by health insurance had a negative effect, which indicated that respondents with acute episodes who were never covered were significantly less likely to go to health facilities compared to those who had always been covered by health insurance. For respondents with chronic episodes, being elderly and having a high adjusted household income had a positive effect, which indicated that elderly respondents were significantly more likely to go to health facilities compared to respondents in early adulthood; and high-income respondents were significantly more likely to go to health facilities than those with low incomes.

## Discussion

The results bring forth three important discussion points about the changes in health-seeking behaviour before and after the implementation of universal health coverage in Indonesia. First, the implementation of UHC had a greater effect for respondents with chronic episodes than for those with acute episodes. The utilisation of public health facilities increased significantly after the implementation of UHC. Private health facility utilisation also increased, but it was not significant among the respondents with acute episodes. The number of respondents who chose no medication or informal care decreased significantly among those with acute and chronic episodes. Second, the proportion of respondents with acute episodes that had never married and chose to go without medication increased from 34.8% to 43.5%. Third, we still found respondents who did not have health insurance after the implementation of UHC. Some people lost their health insurance coverage after the implementation of UHC, and some people had never been covered by health insurance, neither before nor after the implementation of UHC.

### The implementation of UHC had greater effect on respondents with chronic episodes

One would expect healthcare consumers to have a certain preference for one type of care over another. This is especially true for applicants who have prepaid for their services or who are likely to have a personal preference for facility-based care [37–39]. This study showed that after the implementation of UHC, there was an increase in the proportion of respondents who chose formal health services, particularly public health facilities; and there was a decrease in the proportion of respondents who chose no medication or informal care. These findings were, in general, found to be consistent with previous findings, in which the utilisation of public health services was increased and the utilisation of informal care decreased simultaneously after implementation of UHC (or a health insurance program) [40–43]. Of respondents with acute episodes, the implementation of UHC had no significant effect in model 1 but the likelihood of going to a health facility increased significantly after we included the control variables (model 2). This shows that the implementation of UHC is not a dominant factor influencing respondents with acute episodes to go to health facilities. However, the implementation of UHC increased the likelihood that respondents with chronic episodes will go to health facilities for care, even after controlling variables were included (model 2). These results suggest that the implementation of UHC had a greater effect on respondents with chronic episodes than on those with acute episodes. Different results were seen in prior studies in Georgia (2015). Gotsadze et al*.* found that the introduction of Medical Insurance Program (MIP) had greater impact on patients with acute illness than chronic patients. The MIP was effective for increasing the odds of using services for those with acute illnesses, but had almost no effect in terms of increasing the odds that chronic patients would use health services [44].

In our study, the utilisation of public health services increased among people who did not have health insurance and those with high incomes. This may be because the implementation of UHC has contributed to improving the quality of public health services, increasing the likelihood that people would choose to use these public health services. People who did not have health insurance were free from the obligation to use certain services, so they could visit the desired health facilities. Likewise, people with high incomes may be able to afford more expensive private health services, but still, they prefer public health facilities.

In addition, shifting from informal care to public-facility care may improve safety in the use of health care. Before the implementation of UHC, people relied on informal care, and a considerable proportion of informal care might have included self-medication (for example, OTC drugs and traditional/herbal medicine). Such self-medication is often carried out without a doctor's prescription or supervision; therefore, inaccurate self-diagnosis, dosage, and treatment choices could create serious safety issues that could cause harmful effects on health [45]. In this sense, the implementation of UHC may have a positive effect on such safety issues in healthcare utilisation [40].

### Increase in no medication among never-married people

While most of the characteristics of the respondents that we measured showed a decrease in choosing no medication, those who had never married and had acute episodes showed an increase. Unmarried people have less access to resources that can affect utilisation (e.g. health insurance and disposable income) than those who are married, and they may engage in risky health-related behaviour [46–48]. Joung et al*.* found that the relationship between marital status and preference for services utilisation was influenced by confounding factors, mainly level of education [49]. Pandey et al*.* showed that those who married had higher odds of having a recent outpatient visit [46]. In our study, the never-married respondents with acute episodes who chose no medication were characterised as young, highly educated people but with low adjusted household incomes. Their illnesses were not serious, and even though they were insured, they were still choosing no medication (see Supplementary Table 3 for characteristics of respondents with acute episode who chose no medication after the implementation of universal health coverage by marital status). This finding supports a prior evaluation, which showed that about half of the covered people did not use the benefits for outpatient care, and 20% did not use the privileges for inpatient care [24]. With their educational level, they might be better able to decide whether they need to go to formal health services.

### The existence of uninsured people

We still found respondents who did not have health insurance after the implementation of UHC. They were included in the 26.1% of the Indonesian population that remain uninsured, a large proportion of which are employed in the informal workforce, requiring them to pay their own insurance premiums [10, 24, 50]. This group, which the Organisation for Economic Co-operation and Development (OECD) refers to as the ‘missing middle’, earned too much to be eligible for the subsidised coverage offered to the poor and near-poor. The problem of the missing middle is a consequence of current policies [24, 51]. Although the government fully subsidises the premiums of those living in poverty and those living slightly above the poverty line, the ‘missing middle’ remain uncovered by JKN-KIS because of the rapid changes in household welfare and eligibility [52]. In our study, the uninsured people can be divided into two categories: those who lost their coverage after the implementation of UHC and those who never had coverage before or after the implementation of UHC. They were characterised as unemployed with low adjusted household incomes; thus, the possible reason why they were not insured was because they were unable to pay the premiums (see Supplementary Table 4 for characteristics of respondents who did not have health insurance after the implementation of universal health coverage). Several studies have shown that insurance premiums are not the main barrier, but rather the availability of services and a poor understanding of health insurance. Hence, increasing investment in health facilities and campaigns to educate the public about the importance of health insurance is required to expand universal coverage [52].

### Limitation

We acknowledge that this study has several limitations. Firstly, the use of a retrospective cohort study method has the potential to create information bias which certainly affects the results. Providing benchmarking information would improve the accuracy of respondents' recall and a well-trained interviewer may be able to elicit information on underreported events [30]. Secondly, since this study was only conducted in one city, we cannot ensure that the results can be generalized to represent the whole country. However, this study might provide an overview of health-seeking behaviour in Indonesia. Further research with extensive data originating from various regions in Indonesia needs to be carried out to obtain results that are representative of Indonesia. Finally, our questionnaire did not include questions about the reasons why respondents joined JKN-KIS or why they remained uninsured. Further research on the specific topic of uninsured people is needed to provide evidence for developing specific strategies to address these problems.

## Conclusion

There was a change in health-seeking behaviour in the Bandung City community after the implementation of universal health coverage; that is, there was a significant increase in the use of public health facilities. The implementation of UHC appeared to be effective in increasing the odds of using health facilities for those with acute and chronic episodes. The effect seemed greater for those with chronic episodes than acute episodes. However, the implementation of UHC must include other factors to have a significant effect on those with acute episodes. Other factors that had a significant effect on health-seeking behaviour for those with acute episodes include being in middle adulthood, including wage-earning workers, contribution-assistance recipients, and non-wage-earning workers with JKN-KIS membership; never having been covered by health insurance; having a somewhat serious illness; and having very good or excellent current health status. Other factors that had a significant effect on health-seeking behaviour for those with chronic episodes include being elderly, having a high adjusted household income, having been diagnosed with two chronic diseases, and having very good or excellent current health status. Although the implementation of universal health coverage has improved utilisation in public health facilities, the presence of people who are not, or have never been, covered by health insurance, characterised by unemployment and low incomes, is a potential problem that can threaten future improvements in healthcare access and utilisation. Our limitation is that the questionnaire did not include questions about the reasons why respondents joined JKN-KIS or why they remained uninsured. Further research on a larger scale and the specific topic of uninsured people is needed to provide evidence for developing specific strategies to address these problems.

## Supplementary Information

**Additional file 1.** 

**Additional file 2.** 

**Additional file 3.** 

**Additional file 4.**

**Additional file 5.** 

**Additional file 6.** 

## Data Availability

All data generated or analysed during this study are included in this published article and its supplementary information files.

## Abbreviations

UHC: Universal health coverage
JKN-KIS: *Jaminan Kesehatan Nasional-Kartu Indonesia Sehat* (National Health Insurance – Indonesian Healthy Card)
ASKES: *Asuransi kesehatan* (Health insurance)
JAMKESMAS: *Jaminan kesehatan masyarakat* (Community health insurance)
JAMSOSTEK: *Jaminan sosial tenaga kerja* (Social labour security)
ASABRI: *Asuransi sosial angkatan bersenjata Republik Indonesia* (Indonesian armed forces social insurance)
SSAH: Social Security Agency for Health
PBI: *Peserta penerima bantuan iur* (Contribution-assistance recipients)
PPU: *Peserta penerima upah* (Wage-earning workers)
PBPU: P*eserta bukan penerima upah* (non-wage-earning workers)
*Puskesmas*: P*usat kesehatan masyarakat* (public health centre
