# Supplementary material for: Determinant factors behind changes in health-seeking behaviour before and after implementation of universal health coverage in Indonesia
Source: BMC Public Health. 2022 May 12;22:952. doi: 10.1186/s12889-022-13142-8 (PMC9102261; doi:10.1186/s12889-022-13142-8)
Supplement: Supplementary file 5 — Additional file 5. [file 12889_2022_13142_MOESM5_ESM.docx]

**Determinant factors behind changes in health-seeking behaviour before and after implementation of universal health coverage in Indonesia**

Dadan Mulyana Kosasih^1,2^, Sony Adam^2^, Mitsuo Uchida^1^, Chiho Yamazaki^1^, Hiroshi Koyama^1, *^, Kei Hamazaki^1^

^1^Department of Public Health, Graduate School of Medicine, Gunma University, Japan

^2^ Health Office of Bandung City, West Java, Indonesia

**^*^Corresponding author:** Hiroshi Koyama, Department of Public Health Gunma University, 3-39-22 Showa, Maebashi 371-8511, Japan. Telephone: +81272208010 fax: +81272208016,

e- mail: [hkoyama@gunma-u.ac.jp](mailto:hkoyama@gunma-u.ac.jp)
